# Reduced helping intentions are better explained by the attribution of antisocial emotions than by ‘infrahumanization’

**DOI:** 10.1038/s41598-022-10460-0

**Published:** 2022-05-12

**Authors:** Florence E. Enock, Harriet Over

**Affiliations:** Department of Psychology, University of York, York, YO10 5DD UK

**Keywords:** Psychology, Human behaviour

## Abstract

We challenge the explanatory value of one of the most prominent psychological models of dehumanization—infrahumanization theory—which holds that outgroup members are subtly dehumanized by being denied human emotions. Of central importance to this theory is the claim that, to the extent that other people are ‘infrahumanized’, they are less likely to be helped. We examine this hypothesised relationship across four pre-registered and well powered studies. We do not find that attributing all uniquely human emotions to others is positively associated with helping intentions towards them. Instead, we find that attributing prosocial emotions is positively associated with helping intentions and attributing antisocial emotions is negatively associated with helping intentions, regardless of emotion humanness. In our data, what previously appeared to be an association between subtle dehumanization and reduced helping is better explained by the tendency to avoid helping others when we view them negatively.

## Introduction

Discrimination is a pressing global problem. According to the mainstream view, including accounts from social psychology, social neuroscience, philosophy and sociology, outgroup members are vulnerable to harm because they are perceived to be ‘less human’ than the ingroup^1–4^. In these cases, inhibitions against causing outgroup members harm are thought to be eroded, with associated negative behavioural consequences^5–8^. Haslam and Loughnan summarise the consensus opinion by stating that: “dehumanization is important as a psychological phenomenon because it can be so common and yet so dire in its consequences”^5^.

Theories of dehumanization hold that when others are blatantly dehumanized, and thus removed from the category ‘human’, they may fall victim to extreme harm such as genocide and torture^9–11^. Support for this claim comes primarily from the historical record, for example propaganda in which victims are compared to or even described as rats, lice and other animals^9,11,12^. Many researchers in social psychology hold that dehumanization exists along a continuum, from blatant to subtle forms. In subtle forms of dehumanization, outgroup members are thought to be considered somewhat ‘less human’ than ingroup members and to possess uniquely human emotions, traits and other mental states to a lesser extent^1–4^. Support for subtle forms of dehumanization comes primarily from lab-based research using both experimental and correlational designs^5^.

Among the most prominent psychological models of subtle dehumanization is infrahumanization theory^3,4^. This theory proposes that outgroup members are thought to experience uniquely human emotions, such as pride and guilt, to a lesser extent than do ingroup members. The hypothesised intergroup differences in emotion attribution have been reported across a multitude of social contexts including national, racial, religious and political outgroups^13–16^.

According to infrahumanization theory, to the extent that outgroup members are infrahumanized, they are less likely to receive help. Cuddy and colleagues^17^ asked participants to infer emotional states of own race or other race victims after Hurricane Katrina, and whether they intended to help the victims. The less strongly participants attributed uniquely human emotions to outgroup members, the less likely they were to indicate they would help them. Related experimental research in an interpersonal context has shown that participants responded less prosocially when a stranger expressed emotions shared with other animals (e.g., anger)^18^ than when they expressed uniquely human emotions (e.g., disappointment). Follow-up work suggested this effect to be dependent on intergroup context^19^.

Of key importance to infrahumanization theory is the claim that infrahumanization is distinct from intergroup preference because participants ascribe uniquely human emotions that are both positive to experience (e.g., hope, compassion) and negative to experience (e.g., guilt, remorse) more strongly to ingroup than outgroup members^3,4^. Associations between uniquely human emotion ascriptions and helping others have been reported both for positive and negative emotion exemplars^17–19^. That the reported effects are observed for negative human emotions is claimed to be crucial for separating infrahumanization from intergroup preference because it implies that perceiving a group as ‘human’ is different from perceiving them as ‘good’. As Leyens and colleagues (2000, p.189) note in their original hypotheses:

People should more easily associate their ingroup than an outgroup with secondary emotions. This preferential association should be true independent of the valence of the secondary emotions. Indeed, it is the category of secondary emotions as such that is considered typically human. No qualification is made for positive or negative secondary emotions.

Explaining the hypothesised causal connection to helping intentions, Vaes and colleagues (2002, p. 523) suggest:

… an in-grouper who uses secondary emotions has a better chance to induce a prosocial reaction than another in-grouper who uses primary emotions. This result should occur independently of the valence of the emotions. Although helping is facilitated in a positive context … secondary emotions are uniquely human and should as such show their effects over and above the possible impact of valence.

We maintain that evidence claiming to distinguish infrahumanization from intergroup preference may be considerably less convincing than it first appears. Indeed, it appears to rest on a conceptual misunderstanding^20–22^. Emotions are inherently social in nature rather than simply an individual experience^23^. As a result, they can vary both in the extent to which they are positive or negative to experience and in the extent to which they are prosocial or antisocial. We define prosocial emotions as those that reflect the tendency to act in a way that benefits others, displays of which are closely linked to how kind someone is perceived as being. Antisocial emotions, on the other hand, are those that reflect a lack concern for others, displays of which are closely linked to how unkind someone is perceived as being. Emotions such as guilt and remorse are negative to experience but they are not antisocial in character. Rather, they are thought to foster prosocial, reparative responses and individuals who display them following wrongdoing tend to be viewed more positively than those who do not^24–26^.

We suggest that a failure to consider the sociality of the emotions tested has led to a fundamental confound in much previous infrahumanization research. That is, the uniquely human emotions included in stimuli sets are often more prosocial than are the emotions shared with other species. As a result, what appears to be a subtle process of dehumanization may, in fact, be better explained in terms of intergroup preference. Empirically addressing this critique, Enock and colleagues^22^ recently re-examined the central predictions of infrahumanization theory across seven intergroup contexts whilst controlling for emotion sociality. Contrary to the predictions of infrahumanization theory, when emotion sociality was controlled, outgroup members were not denied all of the uniquely human emotions. In these studies, outgroup members were thought to experience prosocial emotions to a lesser extent than ingroup members, but antisocial emotions to a greater extent than ingroup members, regardless of perceived emotion humanness.

This critique has implications for the hypothesised relationship between intergroup emotion attribution and helping. Cuddy and colleagues found that inferences of uniquely human emotions such as grief and guilt predicted intentions to help outgroup members, whilst inferences of emotions shared with animals such as anger and rage did not^17^. The researchers concluded that ‘infrahumanizing’ outgroup members was associated with a reduced likelihood to help them. However, it is also plausible that the more prosocial character of the uniquely human emotions drove this difference. In work by Vaes and colleagues^18,19^ participants may have responded more prosocially to strangers who expressed ‘disappointment’ than ‘anger’ not because disappointment is uniquely human, but because individuals expressing disappointment tend to seem more pleasant than those expressing anger.

We revisit the claim that denying others uniquely human emotions leads to reduced helping. In Study 1, we conceptually replicate the apparent relationship between uniquely human emotion attribution and helping intentions reported in previous research. Following the majority of research in the field, we select a small number of emotion items that are highly representative of each emotion category of interest (i.e., those that are positive or negative to experience and uniquely human or shared with other species) and measure the extent to which participants believe each item is typically felt by ingroup and outgroup members, followed by the extent to which participants are willing to help those ingroup and outgroup members. In Study 2, we select emotions that vary in humanness and sociality from a pretest and show that in our data, the apparent relationship between infrahumanization and reduced helping reported in Study 1 is more likely to reflect the tendency to help others more when we ascribe them prosocial emotions and to help others less when we ascribe them antisocial emotions. In Studies 3 and 4, we provide converging experimental evidence that by our measures, it is the sociality of attributed emotions, rather than the humanness of attributed emotions, that influences intentions to help. Whereas describing individuals (Study 3) and groups (Study 4) as experiencing prosocial emotions increases intentions to help, describing them as experiencing antisocial emotions reduces intentions to help, regardless of whether or not the tested emotions are uniquely human.

## Study 1

### Associations between intergroup emotion inferences and helping intentions

Infrahumanization theory states that the extent to which outgroup members are attributed uniquely human emotions will positively predict people’s willingness to help them, regardless of emotion valence^17^.We aimed to conceptually replicate this apparent relationship. Participants rated the extent to which they thought ingroup or outgroup members experienced emotions that varied in valence (positive or negative to experience) and humanness (uniquely human or shared with other animals) and then rated how likely they would be to donate to a group specific cause.

We employed a religious intergroup context, measuring Christian participants’ responses towards either Christians (ingroup) or Muslims (outgroup). Dehumanization of religious outgroups, including of Muslims by Christians, has been widely reported^13,27,28^.

## Methods

### Ethics

All studies (1–4) were approved by the Ethics Committee at the Department of Psychology, University of York, UK (approval number 819). Informed consent was obtained at the start of each session according to approved ethical procedures. All studies were performed in accordance with the relevant guidelines and regulations.

### Data collection

Data collection for all studies (1–4) took place online and studies were created and administered using Qualtrics (https://www.qualtrics.com). Participants were recruited through Prolific (https://www.prolific.co) and different participants took part in each of the four studies. Participants were compensated at an approximate rate of £7.50 per hour. All studies are pre-registered and are well powered for detecting our effects of interest. Links to pre-registration documents and raw data can be found at: https://osf.io/3rf4m/

### Participants

200 participants completed the experiment. A power analysis using G*power 3.1 found an N of 170 to be sufficient to detect significant effects of each predictor and interaction in the moderated regressions with a medium effect size (f^2^ = 0.15), an alpha of 0.05 and power of 0.95. Participants were all 18 or over. For the intergroup context to be meaningful, participants had to identify as Christian and they had to be fluent in English to ensure they would adequately understand the written stimuli. Participants had to be UK nationals because we developed our emotion stimuli from UK nationals (see pretest in Supplementary Information) and the possibility of cross-cultural differences in perceptions of the emotion terms could not be ruled out^29^. Additionally, participants could only take part if they identified as right wing. This was to maximise our chances of detecting infrahumanization of the Muslim ‘outgroup’ should it occur. Prior work suggests that individuals who identify as right wing are more likely to ‘dehumanize’ others^30^. Finally, as our outcome measure related to food bank donation, we asked participants about their use of food banks after the study was complete. No participants indicated they used a food bank.

In line with our pre-registration, we excluded and replaced 4 participants that failed the attention check. Of the final sample, 106 participants were female and 94 were male, aged from 18 to 80 (Mean age = 43.9, SD = 15.8).

### Materials

#### Emotion stimuli

In testing infrahumanization theory, past research typically selects a small number of emotion items that are highly representative of each emotion category of interest (i.e., uniquely human positive, uniquely human negative, non-uniquely human positive, non-uniquely human negative) and then measures the extent to which participants believe each item is typically felt by ingroup and outgroup members^4,13,14,31^. For example, in measuring the effect of emotion expression on prosocial behaviour, Vaes and colleagues included one or two emotion items to represent each of the emotion categories of interest^18,19^. We follow this standard approach in our conceptual replication and subsequent extensions and select 12 emotion terms in total, three items to represent each condition of interest across our studies.

We chose items from previous research that varied in whether or not they were uniquely human and whether they were positive or negative to experience^17,32^. Table 1 shows the final emotion words included.

**Table 1:** Emotion terms included for each condition in Study 1.

	Positive	Negative
Uniquely human	Admiration	Remorse
	Compassion	Grief
	Empathy	Guilt
All animals	Lust	Rage
	Surprise	Anger
	Desire	Panic

#### Scales

Participants made emotion attributions to the target group by indicating on a sliding scale the extent to which they thought members of the group experienced each of the twelve emotions. Half of the participants rated Christians (the ingroup) and the other half rated Muslims (the outgroup). For each item, participants indicated their response on a sliding scale from *Not at all* (0) to *Very strongly* (100), with the midpoint *Somewhat* (50), though they could not see the numbers. For example, in the ingroup condition, the emotion attribution block began: ‘*You will now be asked to rate the extent to which you think Christians typically feel certain emotions. You will be asked to rate twelve emotions in total. Please read each carefully and answer honestly.’* Then, participants responded to each item, such as ‘How strongly do you think Christians typically feel Admiration?’. The twelve emotion items were randomised and one attention check was included halfway through, such as ‘Please indicate ‘not at all.’

Helping intentions were measured using a simple vignette followed by a single willingness to help scale. The target group was the same as participants made emotion attributions for. The vignette (ingroup version) read:

Recently, there has been a significant increase in the number of people receiving support from a food bank for the first time. Currently, the use of food banks in the UK is predicted to rise even more. How likely do you think it is that you would make a donation (this could be in the form of food, money or time) to a church-run food bank for struggling Christian families?

The outgroup version was identical other than ‘church-run’ became ‘mosque-run’ and ‘Christian families’ became ‘Muslim families’. Helping intentions were measured by the extent to which participants indicated they were likely to make a donation on a sliding scale from *Extremely unlikely* to *Extremely likely*. The unmarked scale was scored from 0 to 100. The emotion attribution scale and helping measure was matched exactly between ingroup and outgroup conditions except for whether the target group was described as Christian or Muslim.

### Procedure

One hundred participants were in the ingroup (Christian) target condition and one hundred different participants were in the outgroup (Muslim) target condition. Other than this, the procedure was the same for all participants. Participants were informed that the study was designed to help us understand the ways in which people ascribe emotions to different groups of individuals and to help us understand how different situations impact on behavioural decisions relating to helping others. They were instructed they would be asked to rate twelve emotion words with regards to how much each one is typically experienced by Christians/Muslims (depending on the group condition). They were told they would then be asked a simple and brief question about how likely they would be to engage in a specific helping behaviour. Once informed consent was obtained, brief demographic (age and gender) questions were asked. Screening for eligibility was through Prolific, though we also confirmed participants met the requirements within the demographic questions. Then, participants were taken through the emotion items followed by the helping intention measure. After this, we asked participants about their personal use of food banks. Finally, participants were debriefed and redirected back to Prolific for payment. Most participants took under ten minutes to complete the study.

### Design and data analysis

For the intergroup emotion attributions, there were four emotion conditions: uniquely human positive, uniquely human negative, shared with other animals positive, and shared with other animals negative. The two group target conditions were Christian (ingroup) or Muslim (outgroup). Following our pre-registered plan, scores for each emotion category were obtained by calculating the mean of the three emotion terms within the category for each participant. For example, a participant’s attribution of uniquely human positive emotion was the mean of their ratings for admiration, compassion and empathy.

Four moderated regressions tested relationships between emotion attributions and helping intentions for each emotion condition, and whether these interacted with target group. The emotion attribution scores for each of the four categories were included as predictors, whilst helping intention was the dependent variable and group target was moderator. In each regression, we were primarily interested in two effects on the dependent variable: emotion attribution and emotion attribution*target group.

## Results

### Emotion attribution and helping regressions

Emotion attribution scores followed the pattern predicted by infrahumanization theory, positive and negative uniquely human emotion ratings were greater for ingroup than for outgroup (see Supplementary Information). Participants indicated greater helping intentions towards ingroup members (M = 66.4, SE = 2.66) than outgroup members (M = 48.7, SE = 3.45), *t*(198) = 4.07, *p* < 0.001, *d* = 0.57.

Before conducting the regression analyses, assumptions of each individual regression model were checked and influential scores were identified and removed using Cook’s distance. This resulted in excluding 10 data points for ingroup target models and 9 data points from outgroup target models. Our final sample of 181 was well powered to detect effects of interest. Target group was coded 0 for outgroup and 1 for ingroup and all predictors were mean-centred before the analysis.

#### Attributions of positive uniquely human emotions and helping

Our first regression showed that the extent to which participants attributed positive uniquely human emotions to others positively predicted the extent to which they were willing to help them, *b* = 0.677 [0.44, 0.92], *t* = 5.62, *p* < 0.001. Participants were also more willing to help ingroup than outgroup members, *b* = 10.55 [2.60, 18.50], *t* = 2.62, *p* = 0.01. The effect of positive human emotion attribution on willingness to help others was not moderated by the group membership of the target, *b* = − 0.364 [− 0.84, 0.11], *t* = − 1.51, *p* = 0.133. The model explained approximately 37% of the variance, *R*^*2*^ = 0.368, *F*(3,178) = 34.6, *p* < 0.001.

#### Attributions of negative uniquely human emotions and helping

Our second regression showed that the extent to which participants attributed negative uniquely human emotions to others also positively predicted the extent to which they were willing to help them, *b* = 0.605 [0.38, 0.83], *t* = 5.35, *p* < 0.001. Participants were also more willing to help ingroup than outgroup members, *b* = 13.61 [5.77, 21.46], *t* = 6.80, *p* < 0.001. The effect of negative human emotion attribution on willingness to help was not moderated by the group membership of the target, *b* = − 0.279 [− 0.73, 0.17], *t* = − 1.23, *p* = 0.219. The model explained about 31% of the variance, *R*^*2*^ = 0.308, *F*(3, 178) = 26.43, *p* < 0.001.

#### Attributions of positive emotions shared with other animals and helping

Our third regression showed that the extent to which participants attributed positive emotions shared with other animals positively predicted helping intentions: *b* = 0.277 [0.02, 0.54], t = 2.11, p = 0.036, though this was a smaller effect than found for associations between uniquely human emotion attribution and helping reported in our first and second regressions. Participants were more willing to help ingroup than outgroup members,* b* = 22.30 [14.17, 30.43], *t* = 5.41, *p* < 0.001. The effect of positive shared emotion attribution on willingness to help was not moderated by the group membership of the target, *b* = − 0.001 [− 0.52, 0.52], *t* = − 0.003, *p* = 0.998. The model explained about 15% of the variance, *R*^*2*^ = 0.151, *F*(3, 178) = 10.56, *p* < 0.001.

#### Attributions of negative emotions shared with other animals and helping

Finally, our fourth regression showed that the extent to which participants attributed negative emotions shared with other animals to others did not predict helping, *b* = 0.033 [− 0.22, 0.29], *t* = 0.26, *p* = 0.798, but participants were again more willing to help ingroup than outgroup members, *b* = 22.04 [12.49, 31.59], *t* = 4.55, *p* < 0.001. The null effect of negative shared emotion attribution on willingness to help was not moderated by the group membership of the target, *b* = 0.410 [− 0.94, 0.91], *t* = 1.61, *p* = 0.110. The model explained about 14% of the variance, *R*^*2*^ = 0.144, *F*(3, 178) = 10.01 *p* < 0.001. We note that if we adjust our threshold for significance to an alpha of 0.006 using a Bonferroni correction for the 8 measures in Study 1 (4 regressions × 2 effects; 0.05/8), our key results remain unchanged, however the association between attributions of positive emotions shared with other animals and helping intentions (regression 3) would be considered only a marginal effect.

**Figure 1:**
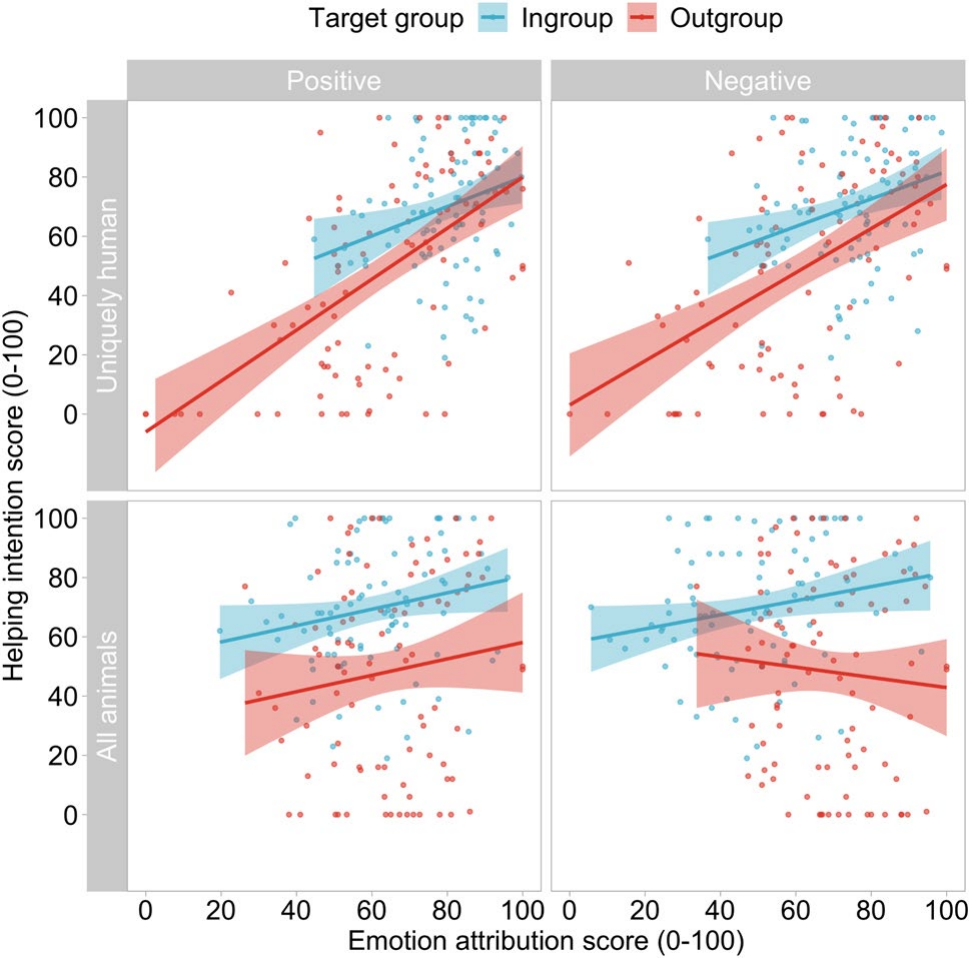
Seemingly in line with infrahumanization theory, the extent to which participants attributed positive and negative uniquely human emotions to outgroup members positively predicted the extent to which they were willing to help them.

We conceptually replicated the apparent relationship between uniquely human emotion attribution and helping intentions predicted by infrahumanization theory and reported in previous empirical research (Fig. 1)^17^. This replication demonstrates that we are well placed to detect evidence of infrahumanization and its behavioural consequences should these effects occur.

## Study 2

### Associations between intergroup emotion inferences and helping intentions—controlling for sociality

In Study 2, we test whether the positive association between uniquely human emotion attribution and helping intentions remains when antisocial emotions are considered. In this design, infrahumanization theory and our alternative view predict different patterns of significance. Infrahumanization theory holds that greater attribution of uniquely human emotions will predict greater helping intentions for outgroup targets regardless of emotion sociality^17^. This should not be the case for emotions shared with other animals. In contrast, in line with our pre-registered prediction, we suggest that willingness to help others will be affected by the sociality of the emotions others are perceived as experiencing, rather than the humanness of the emotions others are perceived as experiencing. As for Study 1, we also expect people will be overall more willing to help ingroup than outgroup members, though this is not central to our research aims.

## Methods

### Participants

200 participants completed the experiment and inclusion criteria were exactly the same as outlined for Study 1. In line with our pre-registration, we excluded and replaced 3 participants that failed the attention check. Of the final sample, 107 participants were female and 93 were male, aged from 19 to 81 (Mean age = 47.7, SD = 15.0). Informed consent was obtained at the start of the session according to approved ethical procedures.

### Materials

#### Development of emotion stimuli

We chose emotion items from pretest data (Supplementary Information, Table S1) that best fit the four emotion categories of interest: uniquely human and prosocial, uniquely human and antisocial, shared with other animals and prosocial, and shared with other animals and antisocial (Table 2). To obtain these items, we chose three words rated as highly prosocial and three rated as highly antisocial from both the most and least uniquely human terms. This follows the standard approach used by past infrahumanization research, which typically selects a small number of items that are highly representative of each emotion category of interest^4,13,14,17,18,31,32^.

**Table 2:** Emotion terms included for each condition in Studies 2–4.

	Prosocial	Antisocial
Uniquely human	Admiration	Contempt
	Humility	Envy
	Optimism	Resentment
All animals	Contentment	Anger
	Joy	Frustration
	Tenderness	Irritation

In line with our manipulations, pretest ratings showed that the uniquely human emotions were rated as significantly more human than the emotions shared with other animals, and the prosocial emotions were rated as significantly more prosocial than the antisocial ones (all *ps* < 0.001). We ensured that humanness was closely matched between the prosocial and antisocial conditions, and that sociality was closely matched between the uniquely human and shared with other animals conditions. This enabled us to measure humanness and sociality as orthogonal and accurately separate effects of each. Further details about stimuli development are reported in Supplementary Information.

### Design

The scales, procedure, design and data analyses exactly followed Study 1 only this time the emotion conditions varied on sociality rather than on valence (Table 2). The four emotion conditions were: uniquely human prosocial, uniquely human antisocial, shared with other animals prosocial, and shared with other animals antisocial.

## Results

### Emotion attribution and helping regressions

Emotion attribution scores did not follow the pattern predicted by infrahumanization theory. Participants ascribed prosocial emotions to ingroup members to a greater extent than to outgroup members, but antisocial emotions to outgroup members to a greater extent than to ingroup members, regardless of emotion humanness (see Supplementary Information). Participants were overall more willing to help ingroup members (M = 70.22, SE = 2.82) than outgroup members (M = 37.68, SE = 3.35), *t*(198) = 7.43, *p* < 0.001, *d* = 1.05.

As for Study 1, four moderated regressions tested for relationships between emotion attributions and helping intentions for each emotion condition, and whether these interacted with target group membership. Influential scores were identified using Cook’s distance and removed, resulting in the exclusion of 12 data points for ingroup target models and 8 data points for outgroup target models. Our final sample of 180 was sufficiently powered to detect effects of interest in the regression analyses. Target group was coded as 0 for outgroup and 1 for ingroup and all predictors were mean-centred before the analysis.

#### Attributions of prosocial uniquely human emotions and helping

The extent to which participants attributed prosocial uniquely human emotions to others positively predicted the extent to which they were willing to help them, *b* = 0.543 [0.28, 0.81], *t* = 4.00, *p* < 0.001. Participants were also more willing to help ingroup than outgroup members, *b* = 31.61 [22.69, 40.54], *t* = 6.99, *p* < 0.001. The effect of prosocial human emotion attribution on willingness to help was not moderated by the group membership of the target, *b* = − 0.359 [− 0.90, 0.18], *t* = − 1.31, *p* = 0.192. The model explained approximately 46% of the variance, *R*^*2*^ = 0.459, *F*(3,176) = 49.77, *p* < 0.001.

#### Attributions of antisocial uniquely human emotions and helping

The extent to which participants attributed antisocial uniquely human emotions to others negatively predicted the extent to which they were willing to help them: *b* = − 0.551 [− 0.76, − 0.35], *t* = − 5.31, *p* < 0.001. Participants were again more willing to help ingroup than outgroup members, *b* = 29.37 [20.85, 37.90], *t* = 6.80, *p* < 0.001. The effect of antisocial human emotion attribution on willingness to help was moderated by the group membership of the target, *b* = 0.531 [0.12, 0.94], *t* = 2.55, *p* = 0.012. Simple slopes analyses showed that the extent of antisocial human attribution negatively predicted willingness to help outgroup targets, *b* = − 0.811 [− 1.08, − 0.54], *t* = − 5.89, *p* < 0.001, but the same trend did not reach significance for ingroup targets, *b* = − 0.280 [− 0.59, − 0.03], *t* = − 1.79, *p* = 0.075. The model explained about 49% of the variance, *R*^*2*^ = 0.490, *F*(3, 176) = 56.28, *p* < 0.001.

#### Attributions of prosocial emotions shared with other animals and helping

The extent to which participants attributed prosocial emotions shared with other animals to others positively predicted the extent to which they were willing to help them, *b* = 0.624 [0.37, 0.88], *t* = 4.78, *p* < 0.001. Participants were more willing to help ingroup than outgroup members, *b* = 30.92 [22.35, 39.49], *t* = 7.12, *p* < 0.001. The effect of prosocial shared emotion attribution on willingness to help was not moderated by the group membership of the target, *b* = − 0.021 [− 0.54, 0.50], *t* = − 0.08, *p* = 0.936. The model explained about 47% of the variance, *R*^*2*^ = 0.467, *F*(3,176) = 51.33, *p* < 0.001.

#### Attributions of antisocial emotions shared with other animals and helping

The extent to which participants attributed antisocial emotions shared with other animals to others negatively predicted the extent to which they were willing to help them, *b* = − 0.378 [− 0.63, − 0.13], *t* = − 3.01, *p* < 0.001. Participants were more willing to help ingroup than outgroup members, *b* = 35.629 [27.06, 44.19], *t* = 8.21, *p* < 0.001. The effect of antisocial shared emotion attribution on willingness to help was moderated by the group membership of the target, *b* = 0.773 [0.23, 1.22], *t* = 2.89, *p* = 0.004. Simple slopes showed that the extent of antisocial shared emotion attribution negatively predicted willingness to help outgroup targets, *b* = − 0.731, [− 1.11, − 0.35], *t* = − 3.79, *p* < 0.001, but did not predict willingness to help ingroup targets, *b* = − 0.009, [− 0.32, 0.30], *t* = − 0.06, *p* = 0.955. The model explained about 43% of the variance, *R*^*2*^ = 0.426, *F*(3, 176) = 43.62, *p* < 0.001. We note that our central results remain unchanged if we correct our p-values using Bonferroni’s adjustment for the multiple tests in Study 2.

**Figure 2:**
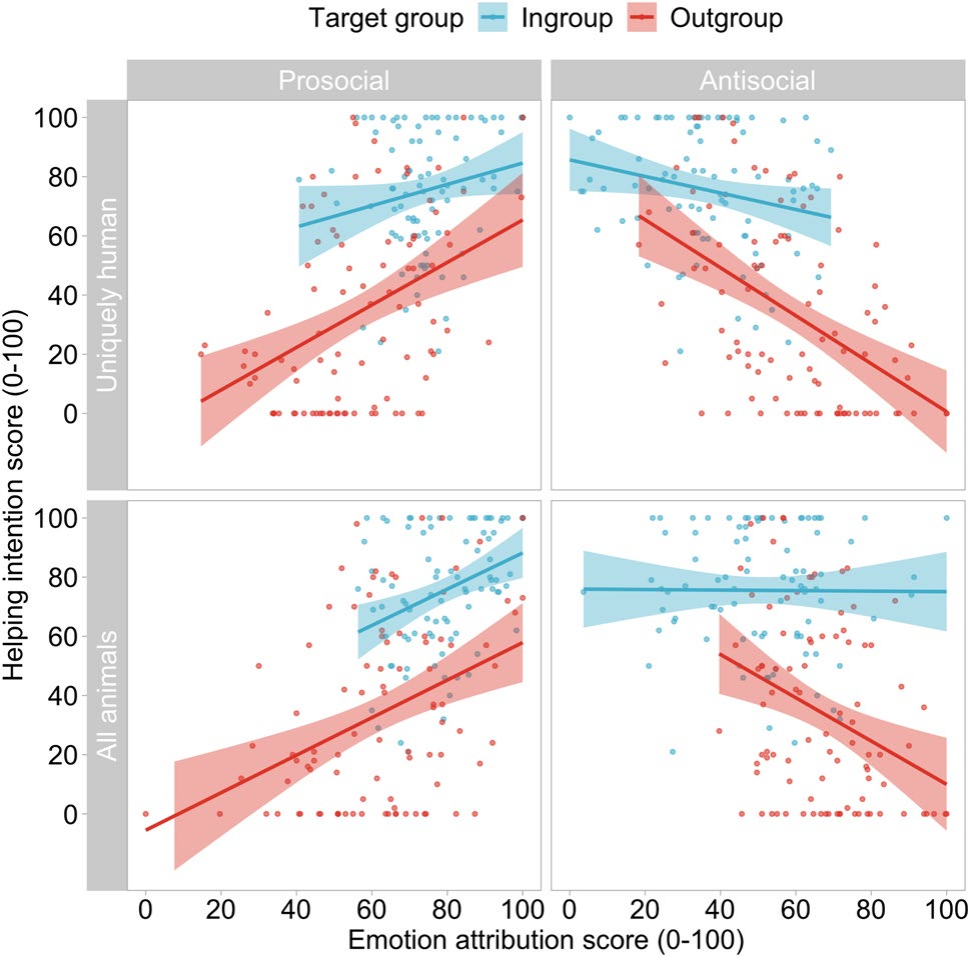
Contrary to predictions of infrahumanization theory, the extent to which participants attributed prosocial emotions positively predicted the extent to which they were willing to help ingroup and outgroup members, but the extent to which participants attributed antisocial emotions negatively predicted the extent to which they were willing to help outgroup (but not ingroup) members, regardless of emotion humanness.

Overall, our results showed that for outgroup targets, helping intentions were positively predicted by prosocial emotion attributions, and negatively predicted by antisocial emotion attributions, both for the uniquely human items and for the items shared with other animals. For ingroup targets, helping intentions were positively predicted by attribution of prosocial emotions, but not by attribution of antisocial emotions. Contrary to infrahumanization theory, in our data, willingness to help outgroup members is affected by the sociality of the emotions they are perceived as experiencing, rather than the humanness of the emotions they are perceived as experiencing (Fig. 2).

## Study 3

### Interpersonal emotion expressions and helping intentions

Previous research has sought to provide causal evidence for the predicted relationship between ‘infrahumanization’ and helping by manipulating the emotions an individual expresses and then measuring the impact on participants’ willingness to help the individual. Past research appears to suggest that participants report greater willingness to help targets who express uniquely human emotions compared to targets who express emotions shared with other animals^18^. We test whether this prediction holds when antisocial emotions are also considered.

Conceptually following Vaes and colleagues^18^, participants read four different emails in which the sender expressed themselves in terms of prosocial uniquely human emotions, antisocial uniquely human emotions, prosocial emotions shared with other animals, or antisocial emotions shared with other animals. Participants then rated how likely they would be to help the sender with a request in the email.

Infrahumanization theory predicts a main effect of humanness such that participants will report they are more willing to help others when they express uniquely human emotions than emotions shared with other animals, regardless of emotion sociality. Summarising this prediction, Vaes and colleagues (2003) note:

Not the expression of any emotion but only the expression of uniquely human emotions should lead to differential reactions [in prosocial responses]… this differential treatment should occur independent of the valence of the secondary emotions. Indeed, because secondary emotions are considered uniquely human altogether, their valence should not matter. (p. 1019).

In contrast, we predict participants will be more willing to help individuals who express prosocial compared to antisocial emotions, regardless of emotion humanness.

## Methods

### Participants

130 participants completed the experiment based on a power analysis using MorePower 6.0.4 that found a minimum N of 126 to be necessary to detect interactions with a medium effect size (partial eta squared 0.06) with an alpha of 0.05 and power of 0.8. Participants were eligible only if they were 18 or over, fluent in English and UK nationals. In line with our pre-registration, we excluded and replaced one participant that failed one or more attention check. Of the final sample, 84 participants were female and 46 were male, aged from 18 to 72 (Mean age = 35.5, SD = 13.9). Informed consent was obtained at the start of the session according to approved ethical procedures.

### Materials

#### Development of emotion stimuli

The same emotion items included for Study 2 represented each of the four emotion categories of interest (Table 2).

#### Vignettes and scales

Conceptually following Vaes and colleagues^18^, we created short email vignettes. In each one, the sender expressed the items from one of the four emotion categories. For example, in the uniquely human prosocial condition, the sender expressed feeling admiration, humility and optimism within the email. At the end of each email, each sender made a simple request for help. There were four senders in total – ‘Alex’, ‘Sam’, ‘Charlie’ and ‘Robin’, names chosen to be gender neutral. Two senders (Alex and Sam) were described as hypothetical colleagues and two (Charlie and Robin) as hypothetical neighbours.

We created the emails such that each of the four senders could express themselves in terms of each of the four emotion categories, giving sixteen possible emails in total. For example, ‘Alex’ describes how their application to be considered for a promotion at work was recently rejected and they ask the recipient to consider nominating them on an online form for special consideration next time. In the uniquely human prosocial version, Alex expresses admiration, humility and optimism. In the uniquely human antisocial version, the email is almost identical but Alex expresses contempt, resentment and envy, and so on for the other emotion conditions. Each participant saw four emails, one of each emotion condition and from different senders. Across participants, email versions and orders were counterbalanced eight ways such that each emotion condition and each sender appeared in each position (first, second, third and fourth) an equal number of times. Full versions of all sixteen emails along with complete counterbalancing information can be found in Supplementary Information.

Participants were asked to indicate on a sliding scale how likely they would be to help the sender with the request in the email, from extremely unlikely to extremely likely. The unmarked sliding scale was scored between 0 and 100. Participants were then asked to indicate how they felt about each sender on a similar scale, this time from extremely negative to extremely positive (see Supplementary Information). Finally, to check attention, there was a simple multiple-choice question about the content of the email.

### Procedure

Participants were informed that the study was designed to help us understand the ways in which different situations can impact on subsequent behaviour decisions relating to helping others. They were instructed that they would be asked to read four short emails, each of which would be from either a hypothetical colleague or neighbour they did not know personally. Participants were asked to try and imagine these were real emails they had received. They were told that after reading the emails, they would be asked a simple, brief question about how likely they would be to respond to the request in each email along with a brief question about attitudes towards each of the senders. Once informed consent was obtained, demographic (age and gender) questions were asked. Screening for eligibility was through Prolific, though we also confirmed participants met the requirements. Then, participants were taken through each email vignette and the corresponding questions. The questions about each individual sender (willingness to help, then attitude, then attention check) were answered directly after reading the relevant email. Finally, participants were debriefed and redirected back to Prolific for payment. Most participants took between five and ten minutes to complete the study.

### Design and data analysis

There were four emotion expression conditions in a 2(humanness) × 2(sociality) within-subjects design. The emotion conditions were: uniquely human prosocial, uniquely human antisocial, shared with other animals prosocial, and shared with other animals antisocial. Helping intentions were measured by the extent to which participants indicated they would be likely to help the senders with the requests in the emails, rated on a scale from 0, *extremely unlikely*, to 100, *extremely likely*. Each participant completed helping intention scales for all four conditions.

A 2 (humanness: uniquely human / shared) × 2(sociality: prosocial / antisocial) repeated measures ANOVA tested for main effects of emotion humanness on helping intention, emotion sociality on helping intention, and for an interaction between the two.

## Results

### Emotion expressions and interpersonal helping intentions

There was a significant effect of sociality on helping intentions, *F*(1, 129) = 169.14, *p* < 0.001, η_p_^2^ = 0.567, with helping scores greater when prosocial (61.1 ± 1.95) compared to antisocial (33.5 ± 1.93) emotions were expressed. There was no significant effect of humanness on helping intentions, *F*(1, 129) = 2.87, *p* = 0.093, η_p_^2^ = 0.022, showing helping scores to be comparable when uniquely human emotions (45.7 ± 1.94) and emotions shared with other animals (48.8 ± 1.80) were expressed (note the slight trend is in the opposite direction to that predicted by infrahumanization theory). There was no significant interaction between sociality and humanness on helping scores, *F*(1, 129) = 0.01, *p* = 0.946, η_p_^2^ < 0.001.

**Figure 3:**
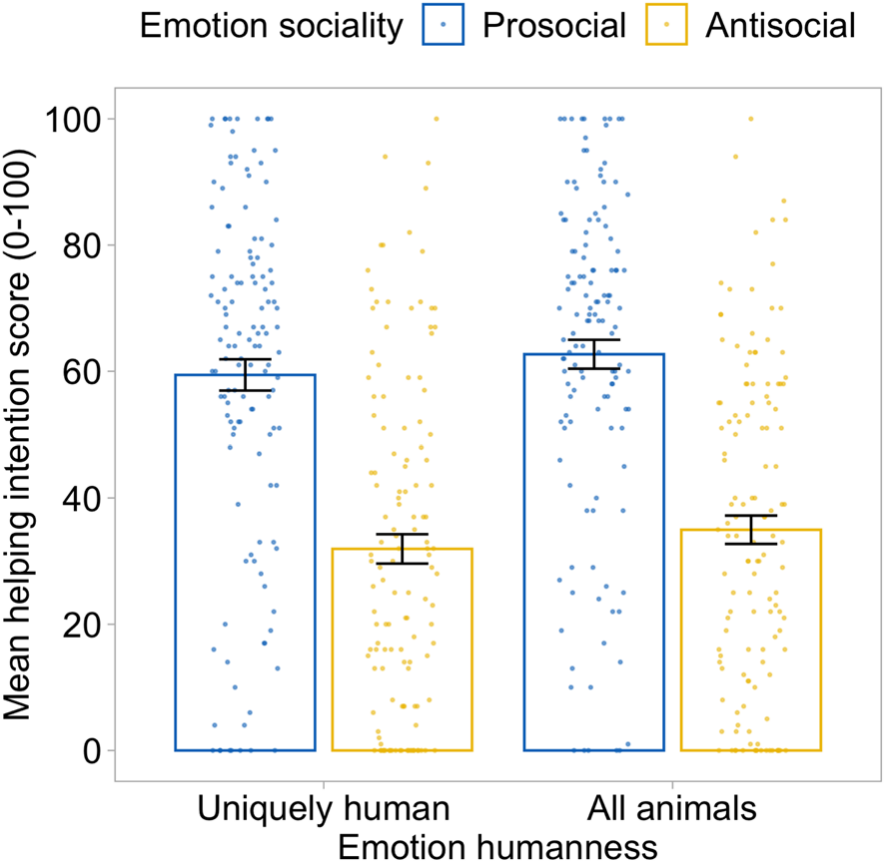
Helping intentions are affected by the sociality, not the humanness, of emotion expressions. Error bars represent standard errors.

Overall, participants were more likely to indicate they would respond to requests for help when the email sender expressed prosocial compared to antisocial emotions, both for the uniquely human emotions and for the emotions shared with other animals. Contrary to infrahumanization, in our data, the humanness of the expressed emotions did not affect helping intentions (Fig. 3). Liking scores followed exactly the same pattern and are reported in Supplementary Information.

## Study 4

### Intergroup emotion expressions and helping intentions

Previous research reports that participants are more likely to help a target individual when they express uniquely human emotions compared to emotions shared with other animals. However, infrahumanization research has also suggested these effects may interact with the group membership of the target^17,19^. Here, we replicate Study 3 in an intergroup context. Participants again read four emails in which the sender expressed themselves in one of the four emotion categories. Half the participants viewed emails from an ingroup sender—a Christian, and half viewed emails from a perceived outgroup sender—a Muslim. Each email again contained a simple request for help.

Infrahumanization theory maintains that an interaction between group membership and emotion humanness will predict helping. We again predict helping intentions will be greater towards others when they express prosocial emotions rather than antisocial emotions, regardless of emotion humanness.

## Methods

### Participants

130 participants completed the experiment based on a power analysis using MorePower 6.0.4 that found a minimum N of 128 to be necessary to detect interactions in the mixed ANOVA with a medium effect size (partial eta squared 0.06) with an alpha of 0.05 and power of 0.8. Inclusion criteria were the same as for Studies 1 and 2 with the additional criteria that participants had to have voted in favour of Brexit in the 2016 referendum. This was to further maximise our chances of detecting bias against the Muslim ‘outgroup’ should it occur^33^. In line with our pre-registration, we excluded and replaced sixteen participants that failed one or more attention check. Of the final sample, 68 participants were female and 62 were male, aged from 18 to 80 (Mean age = 49.9, SD = 14.8). Informed consent was obtained at the start of the session according to approved ethical procedures.

### Materials

The same emotion items as included in Studies 2 and 3 represented the four emotion categories of interest (Table 2). As described for Study 3, we created short email vignettes. In each email, the sender expressed the items from one of the four emotion categories and then made a simple request for help. Each participant saw four emails, one of each emotion condition and from different senders. This time, half of the participants saw emails from Christian (ingroup) senders and half saw emails from Muslim (outgroup) senders. ‘Ingroup’ names were Alex, Sam, Charlie and Robin and ‘outgroup’ names were Nour, Nasim, Tanveer and Majd, all chosen to be gender neutral. The emails always started, ‘Dear Resident’. The ‘ingroup’ senders asked for help with a Christian cause and the ‘outgroup’ senders asked for help with a Muslim cause. The emails were created such that each of the four senders could express themselves in terms of each of the four emotion categories, giving sixteen possible emails × 2 target group senders. The emails were matched exactly between ingroup and outgroup conditions apart from the name and whether the cause related to a Christian or Muslim group. For example, ‘Alex’ describes how the town preservation society has rejected an application for funding to make essential repairs to the church and asks if the recipient would nominate the application for special consideration. The email from ‘Nour’ is identical but the word ‘church’ is replaced with ‘mosque’. In the uniquely human prosocial version, Alex/Nour expresses admiration, humility and optimism. In the uniquely human antisocial version, the email is almost identical but Alex/Nour expresses contempt, resentment and envy, and so on for the other conditions.

As described for Study 3, helping intentions were measured by the extent to which participants were willing to help each sender with the request in the email and attitudes towards each sender were measured on a sliding scale. Multiple-choice questions about the content of the email were included to check attention. Across participants, email versions and orders were counterbalanced eight ways such that each emotion condition and each sender appeared in each position (first, second, third and fourth) an equal number of times. Full versions of all emails along with complete information about counterbalancing can be found in Supplementary Information.

### Procedure

Sixty-five participants were in the ingroup (Christian) group target condition and sixty-five different participants were in the outgroup (Muslim) group target condition. Other than different email vignettes, the procedure was the same for all participants and was identical to that described for Study 3.

### Design and data analysis

There were four (within subject) emotion conditions and two (between subject) target group conditions in a 2(emotion humanness) × 2(emotion sociality) × 2(target group) mixed design. The emotion conditions were: uniquely human prosocial, uniquely human antisocial, shared with other animals prosocial, and shared with other animals antisocial. The two group target conditions were Christian (ingroup) or Muslim (outgroup). Helping intentions were again measured by the extent to which participants indicated they would be likely to help the senders with the requests in the emails. Each participant completed helping intention scales for all four conditions.

A 2(humanness: uniquely human / shared with other animals) × 2(sociality: prosocial / antisocial) × 2(target group: ingroup / outgroup) mixed ANOVA tested for effects of emotion humanness, emotion sociality and target group on helping intentions, along with relevant interactions.

## Results

### Emotion expressions and intergroup helping intentions

There was a significant effect of target group on helping intentions, *F*(1, 128) = 12.67, *p* = 0.001, η_p_^2^ = 0.090, with participants indicating greater willingness to help ingroup (56.0 ± 2.79) than outgroup (41.9 ± 2.79) targets. There was also a significant effect of sociality on helping intentions, *F*(1, 128) = 114.66, *p* < 0.001, η_p_^2^ = 0.473, with participants indicating greater willingness to help targets when they expressed prosocial (64.1 ± 2.30) compared to antisocial (33.9 ± 2.55) emotions. There was no significant effect of humanness on helping, *F*(1, 128) = 1.83, *p* = 0.179, η_p_^2^ = 0.014, showing willingness to help was unaffected by whether targets expressed uniquely human emotions (47.9 ± 2.21) or emotions shared with other animals (50.1 ± 2.10). There was no significant interaction between target group and sociality, *F*(1, 128) = 1.38, *p* = 0.243, η_p_^2^ = 0.011, between target group and humanness, *F*(1, 128) = 0.68, *p* = 0.412, η_p_^2^ = 0.005, nor between sociality and humanness, *F*(1, 128) = 1.60, *p* = 0.208, η_p_^2^ = 0.012. The three-way interaction also was not significant, *F*(1, 128) = 1.05, *p* = 0.307, η_p_^2^ = 0.008.

**Figure 4:**
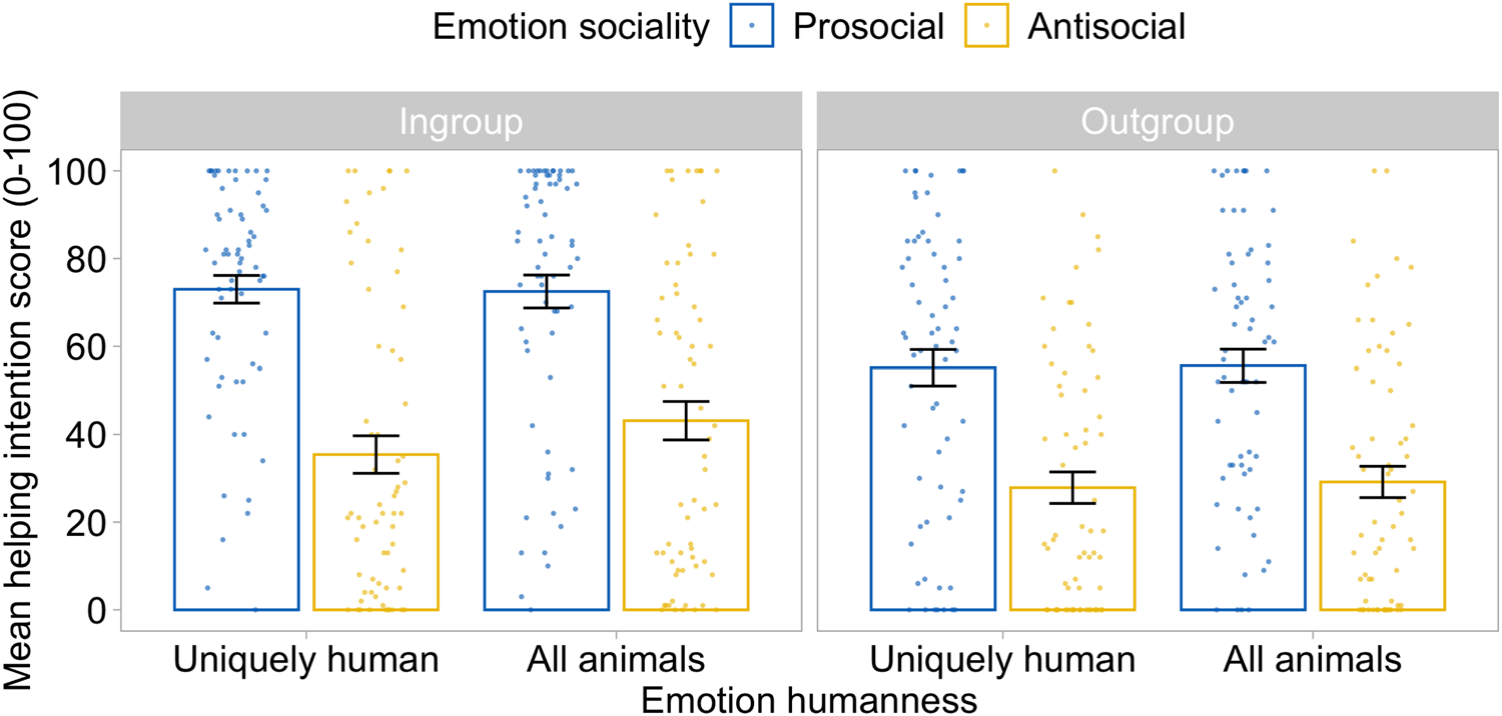
Intergroup helping intentions are affected by the sociality, not the humanness, of emotion expressions. Error bars represent standard errors.

In line with our predictions, participants indicated they were more likely to respond to requests for help when the email sender expressed prosocial compared to antisocial emotions, for both ingroup and outgroup, and for both uniquely human emotions and those shared with other animals. Helping was generally higher for ingroup than for outgroup. Contrary to the predictions of infrahumanization theory, in our data, the humanness of the expressed emotions did not affect intergroup helping intentions (Fig. 4). Liking scores followed exactly the same pattern and are reported in Supplementary Information.

## General discussion

Infrahumanization is hypothesised to be a subtle form of dehumanization whereby outgroup members are denied uniquely human emotions^3,4^. According to infrahumanization theory, to the extent that outgroup members are denied uniquely human emotional experiences, they are less likely to be the recipients of prosocial behaviour^17–19^. Crucial to infrahumanization theory is the claim that: ‘*… seeing someone as lacking human qualities is not the same as derogating them because ‘human’ is not synonymous with ‘good’.’*^34^ However, we contend that previous research on infrahumanization does in fact confound subtle dehumanization with negative evaluation of others and outgroup members. As a result, we suggest that it has so far been impossible to determine whether uniquely human emotion attribution predicts prosocial behaviour or whether previous findings are better explained by the tendency to help others more when we view them positively.

In Study 1, we employed the same emotion stimuli used in prior infrahumanization research^17,32^ and conceptually replicated previously reported effects in a correlational design. This confirmed our methodology and chosen intergroup context was appropriate for probing the theory and testing our alternative explanation.

In Study 2, we interrogated the explanatory value of infrahumanization theory in the same design but this time closely controlled for the sociality of the emotion stimuli. Contrary to the predictions of infrahumanization theory^17^, the extent to which participants attributed uniquely human emotions to outgroup members did not overall positively predict their willingness to help them. Rather, the extent to which participants attributed prosocial emotions to outgroup members positively predicted willingness to help, but the extent to which they attributed antisocial emotions negatively predicted willingness to help.

In Study 3, we experimentally tested whether an individual was more likely to be helped when they expressed uniquely human emotions compared to emotions shared with other animals. Contrary to the predictions of infrahumanization theory^18^, our results showed that helping intentions were higher when individuals expressed prosocial emotions compared to antisocial emotions, regardless of the perceived humanness of the included items.

In Study 4, we further tested the hypothesised causal effect of infrahumanization on helping intentions in an intergroup context. In concordance with Study 3, and contrary to infrahumanization theory, we found that helping intentions were higher for both ingroup and outgroup members when they expressed prosocial compared to antisocial emotions, regardless of the humanness of these items. Our results converge with recent empirical work showing that intergroup biases in trait and emotion attributions are better explained by social preferences than by subtle dehumanization^22,35^.Our results also offer empirical support to broader critiques of the social psychological dehumanization literature, particularly in suggesting that negative behaviours towards others do not necessarily arise when others are seen as less human, but when they are seen in ways that are specific to humans yet antisocial^20,21,36–42^.

It is interesting to consider whether infrahumanization may operate in conjunction with sociality effects such that the two mechanisms are compatible. We find no evidence for this view in our data. Our paradigm was well placed to detect evidence of infrahumanization, in addition to sociality effects, should it occur. First, we conceptually replicated previous findings from the literature in Study 1, demonstrating that our method is sensitive enough to detect evidence of infrahumanization if present. Second, our studies are well powered, offering us a high probability of uncovering effects similar in magnitude to, and indeed smaller than, those reported in previous infrahumanization research. Third, our stimuli are closely matched, meaning that the sociality of the emotions and their humanness were manipulated in an orthogonal fashion, allowing us to detect independent effects of the two variables if they occurred. Despite these factors, we do not find evidence for predictions made by infrahumanization theory in studies 2—4, with our data showing consistent effects of sociality, but not of humanness, on helping intentions.

We acknowledge, however, that that without testing additional intergroup contexts, it remains possible that the effects predicted by infrahumanization theory could sometimes occur in conjunction with (or in the absence of) sociality effects. If future research seeks to investigate the hypothesised causal relation between subtle dehumanization and helping in other intergroup contexts, it will be crucial to implement controls similar to those we include in this paper.

We tested our predictions with one distinct sample – participants identifying as right wing and Christian. We included the criterion of right wing identification to maximise our chances of detecting infrahumanization effects in emotion attribution should they occur^30^. By replicating the effects predicted by infrahumanization theory in Study 1, we show that our methods, intergroup context and participant sample were appropriate and that restriction of our sample to individuals who identified as right wing did not limit our ability to detect the predicted correlations. However, future research would benefit from including a broader sample in addition to a wider range of intergroup contexts, in order to ensure generalisability. Recently, across seven distinct intergroup contexts and participant samples, Enock and colleagues found that outgroup members were not denied uniquely human emotions relative to ingroup members but rather were ascribed prosocial emotions to a lesser extent but antisocial emotions to a greater extent^22^. This converging evidence provides grounds for hypothesising that our present results would generalise to other intergroup contexts and participant samples.

In our data, what previously appeared as an association between infrahumanization and reduced helping is better explained by the tendency to want to help others when we view them positively. While this highlights a serious weakness in previous infrahumanization research, it is far from a comprehensive description of how emotion attribution relates to helping. We note that group specific stereotypes, and their interaction with particular social contexts, are likely to be crucial for understanding how intergroup emotion attributions relates to helping. While examining how stereotypical intergroup emotion attributions relate to helping intentions is an interesting avenue for further work, it was not our goal in this work. Our goal in this research was considerably more modest – we sought to show that apparent evidence for a causal connection between subtle dehumanization and reduced willingness to help others may be better explained by other factors.

In the current work, we focus specifically on one prominent characterisation of subtle dehumanization – infrahumanization – and its hypothesised relation to helping intentions. Further work could revisit previously reported associations between other characterisations of dehumanization and reduced helping intentions. For example, the ‘dual model’ of dehumanization claims that others are less likely to be helped when they are subtly dehumanized by being denied character traits that are uniquely human compared to other animals (‘animalistic dehumanization’) or by being denied character traits that are uniquely human compared to robots (‘mechanistic dehumanization’)^2,43^. However, recent work suggests that the dual model also confounds ‘dehumanization’ with negative evaluation^20,21^. Enock and colleagues reported that when undesirable human-specific characteristics (such as ‘corrupt’ and ‘selfish’) were included in measures of humanness, there was no evidence for either animalistic or mechanistic dehumanization of outgroups as characterised by the dual model^35^. It is possible, then, that previously reported associations between these characterisations of subtle dehumanization and reduced helping may similarly reflect the tendency to help those we evaluate more positively.

We also acknowledge that, following the majority of prior work on infrahumanization, we conceptualised humanness and sociality as categorical variables and included relatively few emotion items for each condition of interest. While this approach is standard in the field^4,17–19^, future work could benefit from testing our predictions against those made by infrahumanization theory in a paradigm that employs more emotion terms and/or manipulates humanness and sociality as continuous predictors. This approach would allow many more emotion items to be included as stimuli^44^.

Understanding the specific ways in which emotion attribution relates to helping others has important applied implications. Infrahumanization theory has become central to many studies examining social cohesion following intergroup conflict^45,46^ and has been used as an outcome measure in interventions designed to improve intergroup relations^47–50^. If psychological research is to effectively inform these sorts of interventions, it is essential it accurately characterises the causes and consequences of the social biases it aims to abate. While here we focus on helping, it would also be beneficial for further work to revisit previously reported links between infrahumanization and explicit harm using experimental designs similar to those developed here.

Overall, the hypothesised process of ‘infrahumanization’ did not explain variation in helping intentions within our studies. We suggest that what appeared to be an association between this hypothesised subtle form of dehumanization and helping in previous research is better explained by the simple tendency to help others more when we view them positively. Our findings provide grounds for further questioning the value of infrahumanization theory within the study of intergroup bias.

## Supplementary Information

Supplementary Information.

## Data Availability

All studies reported in this manuscript were pre-registered and the data is available open access. Link to pre-registration documents and raw data for each study can be found at: https://osf.io/3rf4m/
