# Supplementary material for: Reduced helping intentions are better explained by the attribution of antisocial emotions than by ‘infrahumanization’
Source: Sci Rep. 2022 May 12;12:7824. doi: 10.1038/s41598-022-10460-0 (PMC9098609; doi:10.1038/s41598-022-10460-0)
Supplement: Supplementary file 1 — Supplementary Information. [file 41598_2022_10460_MOESM1_ESM.docx]

Supplementary Information for:

Reduced helping intentions are better explained by the attribution of antisocial emotions than by ‘infrahumanization’

**Authors:** Florence E. Enock^1^ and Harriet Over^1^

^1^Department of Psychology, University of York, York, United Kingdom. YO10 5DD.

*Correspondence to: Florence Enock, Florence.Enock@york.ac.uk.

**1. Pretest: Characterising emotions**

To develop the emotion stimuli used in Studies 2-4, we first measured how 65 emotion terms (in English) are characterised along three separate dimensions: human uniqueness (compared to other animals), valence of experience (negative to positive) and sociality (antisocial to prosocial in character).

**Methods**

**Participants**

200 adults completed the pretest. To be eligible, participants had to be fluent in English so that they adequately understood each emotion term. Additionally, participants had to be UK nationals living in the UK so as to reduce any possible cross-cultural variance in perceptions of the emotion terms ^1,2^. In line with our pre-registration, we excluded and replaced 5 participants that failed one or more attention check. Of the final sample, 128 participants were female and 68 were male, aged from 18 to 71 (Mean age = 35.0, SD = 13.3).

**Stimuli selection**

We chose sixty-five emotion terms and asked participants to rate them on Humanness, Valence and Sociality using three separate sliding scales. We chose many terms that had previously been included in infrahumanization research, along with items from several additional sources within emotion research ^3,4^. The 65 items we included were: Admiration, Adoration, Amusement, Anger, Anxiousness, Awe, Awkwardness, Bitterness, Boredom, Calmness, Compassion, Confusion, Contempt, Contentment, Depression, Disappointment, Disgust, Disillusion, Dissatisfaction, Distress, Embarrassment, Empathy, Envy, Excitement, Fear, Frustration, Gratitude, Grief, Guilt, Happiness, Hate, Hope, Hopelessness, Horror, Humiliation, Humility, Irritation, Jealousy, Joy, Loneliness, Love, Melancholy, Nervousness, Nostalgia, Optimism, Pleasure, Pride, Regret, Relief, Remorse, Resentment, Resignation, Sadness, Schadenfreude, Scorn, Self-satisfaction, Shame, Shyness, Smugness, Spite, Surprise, Sympathy, Tenderness, Triumph, Vengefulness.

**Scales**

Each participant rated the 65 emotion words on three scales. The Humanness scale asked: “Using the slider, please indicate how much the emotion in each of the following questions is experienced by humans compared to other animals (i.e., is this emotion unique to humans?)” The bottom end of the slider corresponded to *Just other animals* and the top to *Just humans*, with the midpoint indicating *Equal to humans and other animals*. On the humanness scale, we were most interested in emotions perceived as shared with other species (scoring close to 50) and those perceived as being only experienced by humans (scoring closer to 100).

The Valence scale asked: “Using the slider, please indicate what you think this emotion is like to experience (i.e., how does it make you feel?).” The bottom end of the slider corresponded to *Extremely negative* and the top end to *Extremely positive*, with the midpoint indicating *Neither negative nor positive*.

The Sociality scale read: “The types of emotions people experience might tell us about their characters. Some emotions may be seen as prosocial, which is the tendency to act in a way that benefits others and is closely linked to how kind or goodhearted someone is. Other emotions may be seen as antisocial, which in this context is the tendency to lack concern for others and is closely linked to how unkind or selfish someone is. Using the slider, please indicate the extent to which you think the experience of each emotion is typically antisocial or prosocial”. The bottom end of the slider corresponded to *Extremely antisocial* and the top end to *Extremely prosocial*, with the midpoint indicating *Neither antisocial nor prosocial*.

Each unmarked scale was scored from 0-100. The three scales were presented in separate blocks on sequential screens and the order of completion was counterbalanced such that one third of participants rated Humanness then Valence then Sociality, one third rated Sociality then Humanness then Valence, and one third rated Valence then Sociality then Humanness. The emotion items within each block were randomised. One attention check per block was included approximately halfway through, such as ‘Please indicate extremely prosocial’. The variable of interest was the rating score given to the emotion terms on each scale.

**Procedure**

Participants were informed that the study was designed to examine the ways in which people understand different emotions. They were instructed that they would be asked to rate emotion terms on the three separate scales. Once informed consent was obtained, brief demographic (age and gender) questions were asked. Screening for eligibility was through Prolific, though we confirmed participants met the nationality and English fluency requirements within the demographic questions. Then, participants were taken through the three scale blocks. Finally, participants were debriefed and redirected back to Prolific for payment. On average, participants took 15 to 20 minutes to complete the study.

**Results and discussion**

Our pretest confirmed emotions are perceived to vary in their humanness, valence and sociality. We present the mean ratings for each emotion on Humanness, Valence and Sociality in Table S1. Broadly replicating previous research, basic emotions such as fear, happiness, sadness, and surprise ^5,6^ featured among those considered shared with other animals. Terms commonly included as uniquely human negative emotions in previous infrahumanization research, such as grief, guilt and shame ^7,8^, did not tend to be perceived as particularly antisocial, though they were considered negative to experience.

Data from our pretest shows the importance in distinguishing between emotions that are negatively valenced and ones that are antisocial and provides grounds for re-evaluating links between infrahumanization and helping intentions. To take one example from previous research, Vaes and colleagues (2002) report that participants were more prosocial towards individuals who expressed uniquely human than non-uniquely human emotions and these findings have been extremely influential in the study of infrahumanization. Sociality scores from our pretest showed that the negative uniquely human items included in the research from Vaes and colleagues (distress and disappointment, mean sociality = 39.1, SD = 14.1) were considered to be significantly more prosocial than the negative non-uniquely human items (irritation and anger, mean sociality = 25.1, SD = 13.2), *t*(199) =13.67, *p* < .001, *d* = .97). We suggest this difference in sociality drove prior effects reported by Vaes and colleagues, rather than differences in humanness.

**Table S1 | Pretest results. Emotion terms scored from highest to lowest along dimensions of Humanness, Valence, and Sociality.**

| **Humanness** | | | **Valence** | | | **Sociality** | | |
| --- | --- | --- | --- | --- | --- | --- | --- | --- |
| *Emotion* | *M* | *SE* | *Emotion* | *M* | *SE* | *Emotion* | *M* | *SE* |
| Nostalgia | 89.6 | 1.03 | Love | 93.0 | 0.80 | Love | 87.9 | 0.97 |
| Smugness | 83.2 | 1.29 | Happiness | 92.5 | 0.83 | Compassion | 86.3 | 1.09 |
| Regret | 83.1 | 1.20 | Joy | 91.7 | 0.90 | Empathy | 84.3 | 1.44 |
| Bitterness | 82.2 | 1.32 | Pleasure | 89.9 | 0.85 | Gratitude | 82.2 | 1.04 |
| Awkwardness | 81.7 | 1.23 | Excitement | 85.9 | 0.97 | Sympathy | 81.9 | 1.18 |
| Embarrassment | 81.3 | 1.26 | Hope | 83.5 | 0.96 | Happiness | 81.1 | 1.10 |
| Spite | 80.3 | 1.33 | Optimism | 83.3 | 1.04 | Joy | 78.2 | 1.10 |
| Disillusion | 79.9 | 1.36 | Triumph | 81.7 | 1.20 | Optimism | 77.4 | 1.15 |
| Optimism | 79.8 | 1.34 | Gratitude | 81.6 | 1.04 | Admiration | 77.2 | 1.03 |
| Scorn | 79.4 | 1.46 | Relief | 80.7 | 1.10 | Hope | 76.4 | 1.11 |
| Humility | 78.7 | 1.37 | Amusement | 80.5 | 1.07 | Tenderness | 75.9 | 1.40 |
| Contempt | 78.1 | 1.34 | Compassion | 80.4 | 1.18 | Excitement | 73.6 | 1.16 |
| Resentment | 77.9 | 1.30 | Calmness | 79.3 | 1.17 | Adoration | 72.9 | 1.24 |
| Guilt | 77.5 | 1.29 | Adoration | 78.3 | 1.20 | Pleasure | 70.8 | 1.21 |
| Humiliation | 77.4 | 1.34 | Contentment | 76.7 | 1.60 | Calmness | 69.7 | 1.28 |
| Hate | 77.2 | 1.38 | Admiration | 76.7 | 0.97 | Humility | 68.2 | 1.62 |
| Remorse | 76.8 | 1.33 | Self satisfaction | 75.0 | 1.42 | Amusement | 67.9 | 1.23 |
| Schadenfreude | 76.8 | 1.66 | Tenderness | 74.2 | 1.44 | Awe | 66.8 | 1.16 |
| Hopelessness | 75.6 | 1.35 | Pride | 72.2 | 1.47 | Contentment | 65.6 | 1.42 |
| Disgust | 75.3 | 1.35 | Awe | 72.1 | 1.22 | Relief | 62.8 | 1.13 |
| Hope | 75.0 | 1.36 | Empathy | 71.7 | 1.40 | Triumph | 62.8 | 1.31 |
| Shame | 74.8 | 1.36 | Nostalgia | 66.6 | 1.35 | Nostalgia | 62.4 | 1.17 |
| Vengefulness | 74.7 | 1.43 | Sympathy | 64.3 | 1.43 | Surprise | 61.8 | 1.00 |
| Self satisfaction | 73.7 | 1.38 | Surprise | 61.9 | 1.13 | Remorse | 59.3 | 1.64 |
| Resignation | 73.5 | 1.39 | Humility | 58.5 | 1.70 | Pride | 58.4 | 1.64 |
| Awe | 72.8 | 1.37 | Smugness | 40.8 | 1.68 | Self satisfaction | 48.4 | 1.62 |
| Admiration | 72.3 | 1.34 | Schadenfreude | 40.4 | 1.66 | Regret | 47.6 | 1.36 |
| Envy | 72.2 | 1.36 | Shyness | 38.2 | 1.06 | Grief | 46.3 | 1.46 |
| Sympathy | 72.0 | 1.27 | Confusion | 35.1 | 1.10 | Confusion | 45.6 | 0.98 |
| Dissatisfaction | 71.4 | 1.36 | Boredom | 33.7 | 1.10 | Guilt | 45.3 | 1.48 |
| Disappointment | 70.9 | 1.24 | Remorse | 32.8 | 1.64 | Melancholy | 42.3 | 1.30 |
| Gratitude | 70.0 | 1.34 | Melancholy | 32.6 | 1.52 | Embarrassment | 41.8 | 1.17 |
| Pride | 69.7 | 1.48 | Resignation | 32.4 | 1.19 | Boredom | 41.5 | 1.29 |
| Empathy | 69.7 | 1.32 | Awkwardness | 30.9 | 1.03 | Nervousness | 41.0 | 1.10 |
| Horror | 69.2 | 1.37 | Nervousness | 30.7 | 1.21 | Sadness | 40.3 | 1.27 |
| Melancholy | 69.1 | 1.27 | Contempt | 29.7 | 1.88 | Shame | 40.1 | 1.48 |
| Relief | 69.1 | 1.23 | Disillusion | 25.5 | 1.09 | Distress | 40.1 | 1.23 |
| Compassion | 66.9 | 1.20 | Embarrassment | 24.7 | 1.22 | Anxiousness | 39.6 | 1.15 |
| Depression | 66.1 | 1.23 | Irritation | 24.5 | 1.05 | Fear | 39.6 | 1.24 |
| Adoration | 65.6 | 1.27 | Frustration | 24.1 | 1.16 | Resignation | 39.1 | 1.10 |
| Triumph | 65.1 | 1.23 | Dissatisfaction | 24.1 | 1.06 | Shyness | 38.9 | 1.13 |
| Jealousy | 63.3 | 1.17 | Regret | 22.8 | 1.24 | Disappointment | 38.2 | 1.11 |
| Amusement | 62.5 | 1.25 | Disappointment | 22.6 | 1.07 | Awkwardness | 38.0 | 1.30 |
| Anxiousness | 61.8 | 1.14 | Anxiousness | 22.3 | 1.31 | Horror | 37.1 | 1.31 |
| Shyness | 61.8 | 1.20 | Envy | 21.0 | 1.16 | Disillusion | 35.8 | 1.12 |
| Frustration | 61.5 | 1.12 | Scorn | 20.8 | 1.12 | Hopelessness | 35.3 | 1.28 |
| Boredom | 61.5 | 1.13 | Guilt | 18.8 | 1.18 | Dissatisfaction | 34.7 | 1.17 |
| Irritation | 60.3 | 1.08 | Vengefulness | 17.8 | 1.29 | Frustration | 34.5 | 1.23 |
| Tenderness | 59.2 | 1.12 | Disgust | 17.6 | 1.11 | Loneliness | 33.9 | 1.44 |
| Anger | 59.1 | 1.02 | Sadness | 17.5 | 1.24 | Humiliation | 33.3 | 1.45 |
| Nervousness | 58.7 | 1.07 | Loneliness | 17.1 | 1.23 | Depression | 31.0 | 1.50 |
| Love | 58.6 | 0.99 | Shame | 17.0 | 1.13 | Schadenfreude | 29.8 | 1.57 |
| Contentment | 58.4 | 1.27 | Jealousy | 17.0 | 1.03 | Disgust | 29.1 | 1.29 |
| Grief | 57.7 | 0.83 | Resentment | 16.5 | 1.04 | Smugness | 28.7 | 1.35 |
| Confusion | 57.5 | 0.99 | Anger | 16.3 | 1.16 | Irritation | 28.5 | 1.05 |
| Surprise | 57.4 | 1.05 | Bitterness | 16.2 | 1.09 | Contempt | 28.0 | 1.68 |
| Joy | 56.0 | 0.92 | Horror | 16.0 | 1.27 | Envy | 23.5 | 1.28 |
| Sadness | 55.7 | 0.76 | Fear | 15.8 | 1.11 | Anger | 21.7 | 1.15 |
| Calmness | 55.6 | 0.98 | Spite | 15.3 | 1.15 | Scorn | 21.2 | 1.15 |
| Loneliness | 55.1 | 0.84 | Humiliation | 15.0 | 1.16 | Resentment | 21.0 | 1.17 |
| Pleasure | 55.0 | 0.84 | Grief | 15.0 | 1.35 | Jealousy | 19.6 | 1.24 |
| Happiness | 54.3 | 0.67 | Hopelessness | 15.0 | 1.22 | Bitterness | 19.3 | 1.11 |
| Excitement | 54.2 | 0.93 | Distress | 13.2 | 1.06 | Spite | 16.0 | 1.24 |
| Distress | 51.1 | 0.73 | Depression | 8.8 | 1.08 | Vengefulness | 15.4 | 1.29 |
| Fear | 51.1 | 0.71 | Hate | 8.4 | 0.92 | Hate | 11.2 | 1.07 |

Mean scores (M) and standard error of the mean (SE) are presented alongside each word. Respective to each scale, 100 indicated the emotion was highly unique to humans / extremely positive to experience / extremely prosocial. 0 indicated the emotion was unique to other animals / extremely negative to experience / antisocial. 50 indicated the emotion applied equally to humans and other animals / neither positive nor negative to experience / neither prosocial nor antisocial. While some emotions were rated similarly on sociality and valence (love was rated as highly positive to experience and highly prosocial), others were rated orthogonally (grief was rated negative to experience but not as antisocial).

**2. Stimuli development for Studies 2-4**

We chose emotion items from our pretest data (Table S1) that best fit the four emotion categories of interest – uniquely human prosocial, uniquely human antisocial, shared with other animals prosocial and shared with other animals antisocial (see Table 2, main text). To obtain these items, we chose three words rated as highly prosocial (scoring towards 100 on the sociality scale) and three rated as highly antisocial (scoring towards 0 on the sociality scale) from both the most and least uniquely human terms. We were interested in finding emotions perceived as shared with other animals (scoring close to 50 on humanness) and those perceived as being more typically experienced only by humans (scoring closer to 100 than 50 on humanness). In the following four t-tests we report, we correct for multiple comparisons using bonferroni’s adjustment by employing an alpha of .01.

In line with our experimental manipulations, paired t-tests showed that for humanness, the uniquely human emotions (76.5 ±.93) were rated as significantly more human than emotions shared with other animals (59.1 ±.66), *t*(199) = 20.83, *p* <.001, *d* = 1.47. On sociality, the prosocial words (73.8 ±.74) were rated as significantly more prosocial than the antisocial words (26.2 ±.80), *t*(199) = 35.63, *p* <.001, *d* = 2.52. We ensured that humanness ratings were closely matched between the prosocial and antisocial conditions and that sociality ratings were closely matched between the uniquely human and shared with other animals conditions. Paired t-tests found that humanness ratings did not significantly differ overall between prosocial (67.4 ±.79) and antisocial (68.2 ±.80) terms, *t*(199) = .98, *p* =.326, *d* = 0.07. This was supported by a Bayes factor in favour of the null model, BF_01_ = 7.86 and ensured that humanness was adequately controlled across the two levels of sociality. Similarly, sociality ratings were comparable between uniquely human emotions (49.2 ±.49) and those shared with other animals (50.7 ±.54), t(199) = 2.21, p =.028, d = 0.16. This was supported by a Bayes factor in favour of the null model, BF_01_ = 1.18. This ensured we could separate effects of sociality from ones of humanness.

We match our emotions for sociality across the two dimensions of humanness and we match our emotions for humanness across the two dimensions of sociality so that we can measure sociality and humanness as orthogonal and test fairly for the effects of each, without either being confounded by the other.

**3. Supplementary results: Intergroup bias in emotion attributions in Studies 1 and 2**

**Study 1**

A 2(target group: ingroup/outgroup) x 2 (humanness: uniquely human/shared with other animals) x 2 (valence: positive/negative) mixed ANOVA tested for intergroup bias in emotion attributions. There was a three-way interaction between target group, humanness and valence, *F*(1, 198) = 19.32, *p* <.001, *ηp²*= .089. Pairwise comparisons showed that uniquely human attributions were greater for ingroup than outgroup, both for positive and negative emotions (both *ps* < .001). However, for positive emotions shared with other animals, there was no difference in ratings between ingroup and outgroup (*p* = .227) and for negative emotions shared with other animals, ratings were higher for outgroup than for ingroup (*p* <.001) (Figure S1). These results replicate the pattern predicted by infrahumanization theory ^7,7–9^.

**
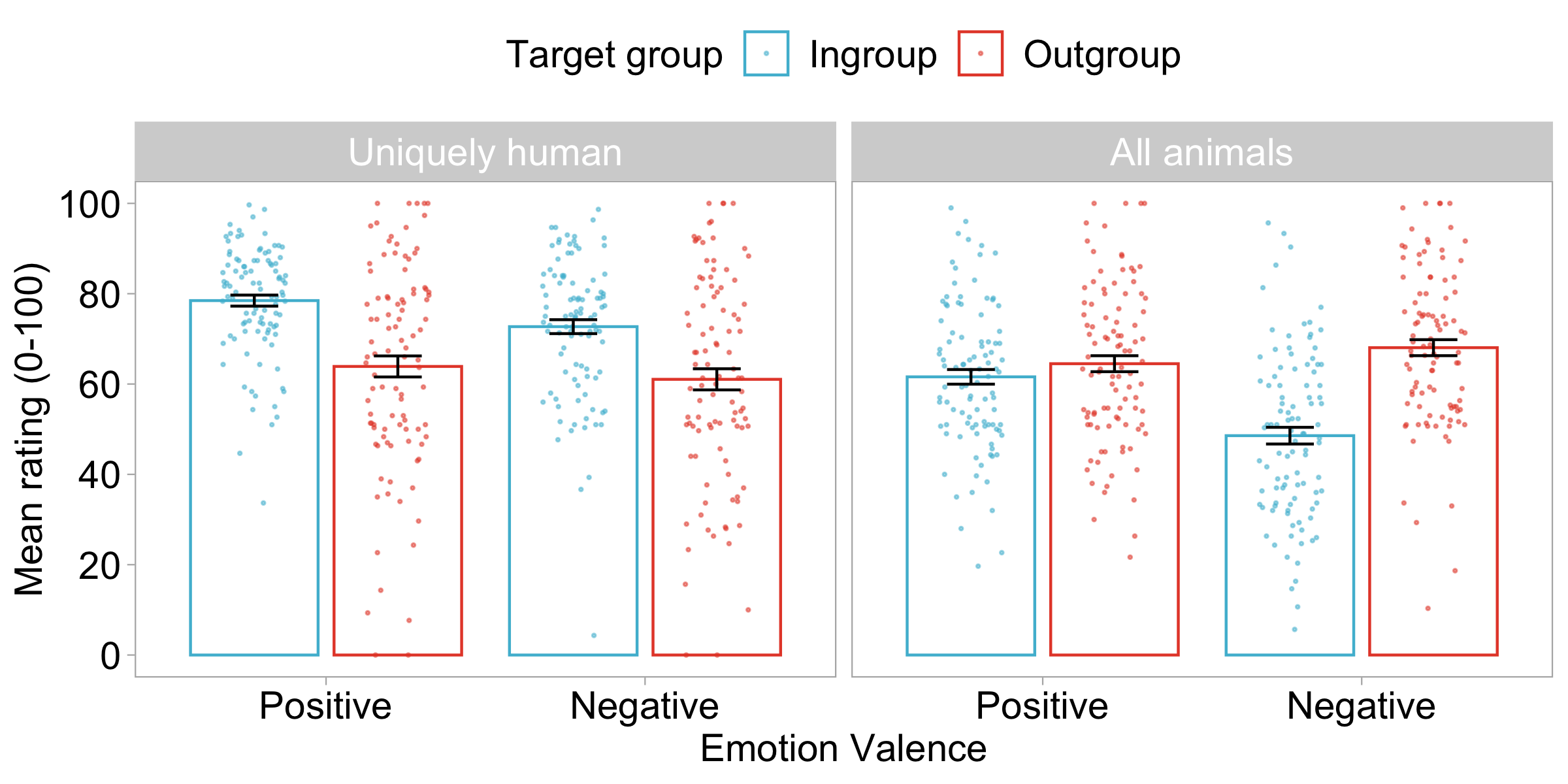
**

**Figure S1.** Seemingly in line with infrahumanization theory, participants ascribed positive and negative uniquely human emotions more strongly to the ingroup than the outgroup, but this was not the case for emotions shared with other animals.

**Study 2**

A 2(target group: ingroup/outgroup) x 2 (humanness: uniquely human/shared with other animals) x 2 (sociality: prosocial/antisocial) mixed ANOVA tested for intergroup bias in emotion attributions. There was a three-way interaction between target group, humanness and valence, *F*(1, 198) = 6.27, *p* =.013, ηp²= .031. Pairwise comparisons showed that prosocial emotion attributions were greater for ingroup than outgroup whilst antisocial emotion attributions were greater for outgroup than ingroup, both for uniquely human terms and for those shared with animals (Figure S2). The infrahumanization pattern observed in Study 1 was no longer present in our data when sociality was controlled.

**
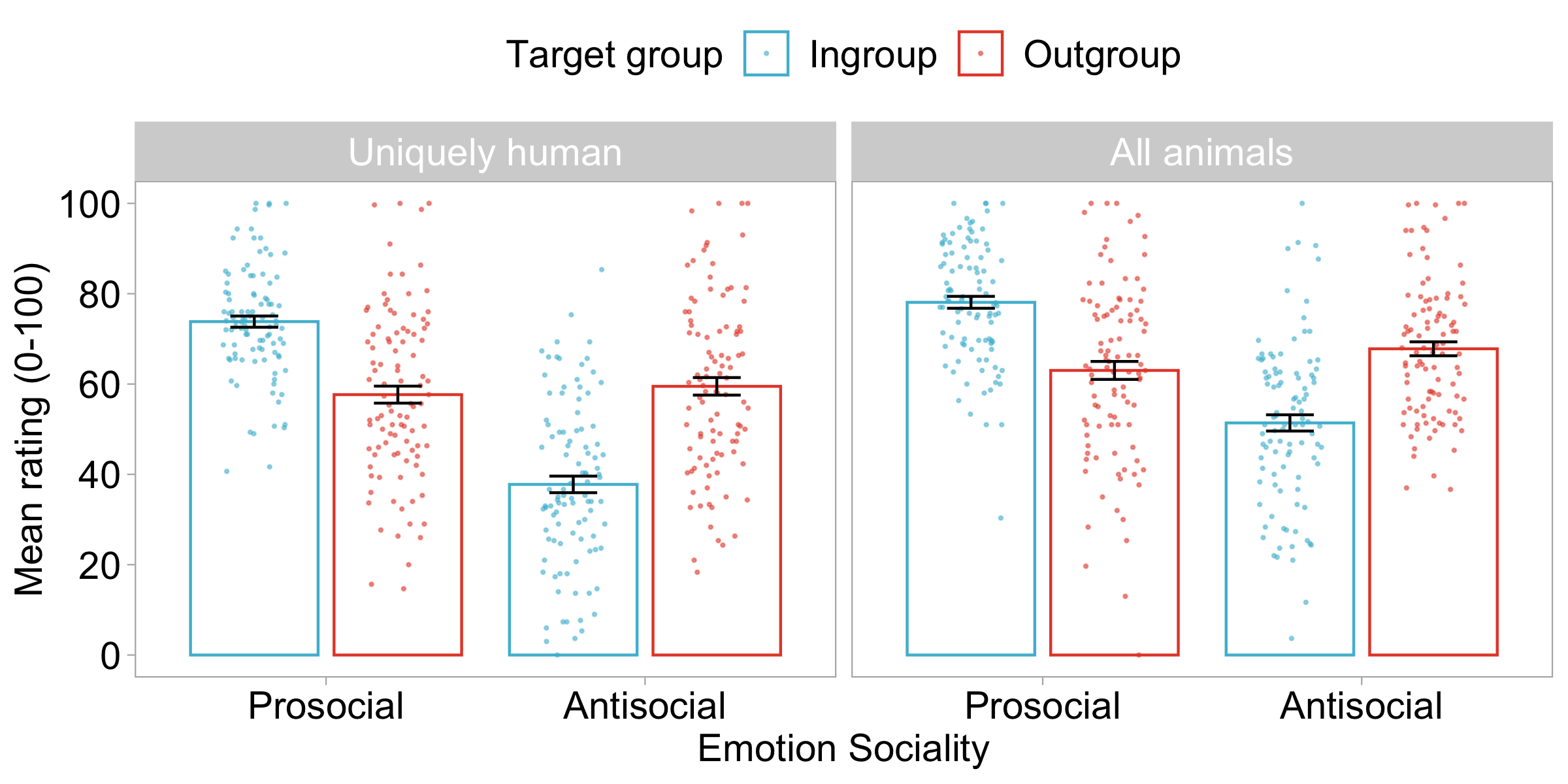
**

**Figure S2.** Contrary to infrahumanization theory, when emotion sociality was controlled, participants ascribed prosocial emotions more strongly to the ingroup than the outgroup and antisocial emotions more strongly to the outgroup than the ingroup. This pattern held for items that were uniquely human and for items that were shared with other animals.

**4. Supplementary results: Emotion expressions and liking scores in Studies 3 and 4**

**Study 3**

As well as our main analysis, we measured differences in how participants felt about the senders across the four emotion conditions with an additional 2 (humanness: uniquely human / shared) x2(sociality: prosocial / antisocial) repeated measures ANOVA on liking scores. Similar to the pattern observed for helping scores (main text), there was a significant effect of sociality on liking, *F*(1, 129) = 248.31, *p* <.001, η_p_^2^= .658, with liking scores greater when prosocial (64.9 ±1.67) compared to antisocial (34.7 ±1.67) emotions were expressed. There was no significant effect of humanness on liking, *F*(1, 129) = 1.61, *p* =.206, η_p_^2^= .012, showing liking scores to be comparable whether uniquely human emotions (48.8 ±1.71) or those shared with other animals (50.9 ±1.49) were expressed. There was no significant interaction between sociality and humanness on liking, *F*(1, 129) = .39, *p* =.533, η_p_^2^= .003.

**Study 4**

Differences in how participants felt about the senders across the conditions were measured with a 2 (humanness: uniquely human / shared with other animals) x2(sociality: prosocial / antisocial) x2(target group: ingroup / outgroup) mixed ANOVA on liking scores. Again similar to the pattern observed for helping scores (main text), there was a significant effect of target group on liking, *F*(1, 128) = 4.37, *p* =.039, η_p_^2^= .033, with liking overall greater for ingroup (58.1 ±1.99) than outgroup (52.2 ±1.99). There was also a significant effect of sociality on liking, *F*(1, 128) = 191.93, *p* <.001, η_p_^2^= .600, with liking greater when prosocial (74.1 ±1.71) compared to antisocial (36.1 ±2.20) emotions were expressed. There was no significant effect of humanness on liking, *F*(1, 128) = 1.01, *p* =.317, η_p_^2^= .008, showing liking scores to be comparable when uniquely human emotions (54.4 ±1.60) and emotions shared with other animals (55.9 ±1.60) were expressed. There was no significant interaction between target group and sociality, *F*(1, 128) = 1.86, *p* =.175, η_p_^2^= .014, nor between target group and humanness, *F*(1, 128) = .19, *p* =.665, η_p_^2^= .001. However, there was a significant interaction between sociality and humanness, *F*(1, 128) = 10.53, *p* =.001, η_p_^2^= .076. For expressions of prosocial emotions, there was no difference in liking scores between uniquely human and shared emotions (*p* = .104). For antisocial emotions, liking scores were significantly greater for the shared than the uniquely human emotions (*p* = .006). The three-way interaction was not significant, *F*(1, 128) = .18, *p* =.670, η_p_^2^= .001. Overall, participants liked the senders more when they expressed prosocial compared to antisocial emotions, both for uniquely human emotions and those shared with other animals, and both for ingroup and outgroup targets. Liking was generally higher for ingroup than for outgroup.

**5. Full vignettes and counterbalancing information for Study 3**

Helping intentions were measured by the extent to which participants were willing to help each sender with the request in the email. The helping requests were all designed to be low-cost and fitting with a request from a stranger, such as signing a petition or endorsing a new business online.

**a.**

*Dear colleague, 
I am writing to let you know that my application to be considered for a promotion within the company was recently rejected. I have worked here for many years now and cannot help but feel great* ***admiration / contempt / tenderness / anger*** *for/towards the company. I feel immensely* ***humbled / resentful / content / frustrated*** *by/about/with how I’ve been treated so far, and I’m of course extremely* ***optimistic / envious / joyful / irritated*** *for/of/towards colleagues who have been more successful than I in the promotions round. I would like to ask you to consider nominating me on the online form for special consideration next time - this can remain anonymous if you wish and it would mean I had a higher chance of success for my next application. 
Kind Regards, 
Alex*

**b.**

*Dear colleague, 
I would like to let you know that I have recently had my hours cut back at work and because of this I am launching a small consultancy side business. I feel immensely* ***humbled / resentful / content / anger*** *by/about/with this new situation. Other similar businesses have recently been started and I’m extremely* ***optimistic / envious / joyful / frustrated*** *about their high chance at success. I feel great* ***admiration / contempt / tenderness / irritation*** *for all small businesses in the current climate and I’m writing to ask if you’d consider endorsing my business on my professional business online profile. This could be completely anonymous and would likely make a tremendous difference in my project’s initial success. 
Kind Regards, 
Sam*

**c.**

*Dear neighbour, 
I would like to draw your attention to a recent decision made by the council to reject my application for planning permission to develop a block of flats. I feel immense(ly)* ***humble / resentful / tenderness / anger*** *about (the difficulty of) this decision, but the plans would have no direct impact on anyone else. I feel great* ***admiration / contempt / contentment / frustration*** *for/with the way the local community is run and I’m extremely* ***optimistic / envious / joyful / irritated*** *for/of/that other people (who) have approval to develop their properties. I would like to ask if you would sign an online petition to encourage the council to reconsider my application? This could be completely anonymous and would likely make a huge difference to the application. 
Kind Regards, 
Charlie*

**d.**

*Dear neighbour,*

*I am writing to tell you that I am planning to enter my personal travel and photography blog into a national competition. Last year I was just beaten to the shortlist and felt immensely* ***humble / resentful / content / frustrated*** *about the decision, given the competition. I felt extreme(ly)* ***optimistic / envious / joyful / anger*** *for/of/towards those that had more success than I. Seeing the quality of entries this year makes me feel great* ***admiration / contempt / tenderness / irritation*** *for the blogging community. With this in mind, I would like to ask you if you’d consider up-voting my blog on the website of entries. This would be completely anonymous and would greatly increase its prominence on the site and my chance of success.*

*Kind regards,*

*Robin*

**Counterbalancing of vignettes for Study 3:**

Each participant saw four emails, one of each emotion condition and from different senders. Across participants, email versions and orders were counterbalanced eight ways such that each emotion condition and each sender appeared in each position (first, second, third and fourth) an equal number of times as shown in Table S2.

**Table S2: Vignette counterbalancing in Study 3.** Columns show version and order of email vignettes that participants saw in each counterbalance condition.

|  | C1 | C2 | C3 | C4 | C5 | C6 | C7 | C8 |
| --- | --- | --- | --- | --- | --- | --- | --- | --- |
| V1 | UH Prosocial Alex (a) | NonUH Prosocial Robin (d) | NonUH Antisocial Sam (b) | UH Antisocial Charlie (c) | UH Prosocial Charlie (c) | NonUH Prosocial Sam (b) | NonUH Antisocial Robin (d) | UH Antisocial Alex (a) |
| V2 | UH Antisocial Sam (b) | NonUH Antisocial Charlie (c) | UH Prosocial Charlie (c) | NonUH Prosocial Sam (b) | UH Antisocial Robin (d) | NonUH Antisocial Alex (a) | UH Prosocial Alex (a) | NonUH Prosocial Robin (d) |
| V3 | NonUH Prosocial Charlie (c) | UH Prosocial Sam (b) | UH Antisocial Robin (d) | NonUH Antisocial Alex (a) | NonUH Prosocial Alex (a) | UH Prosocial Robin (d) | UH Antisocial Sam (b) | NonUH Antisocial Charlie (c) |
| V4 | NonUH Antisocial Robin (d) | UH Antisocial Alex (a) | NonUH Prosocial Alex (a) | UH Prosocial Robin (d) | NonUH Antisocial Sam (b) | UH Antisocial Charlie (c) | NonUH Prosocial Charlie (c) | UH Prosocial Sam (b) |

C is counterbalance number and V is vignette presentation. UH refers to uniquely human emotions. NonUH refers to non-uniquely human emotions, which we also call ‘shared with other animals’.

**6. Full vignettes and counterbalancing information for Study 4**

**a.**

*Dear resident,*

*I am writing to let you know that our application to the town preservation society to be considered for funding to make essential repairs to our* ***church / mosque*** *was recently rejected. We have worked within the town for many years now and I cannot help but feel great* ***admiration / contempt / tenderness / anger*** *for/towards the preservation society. I feel immensely* ***humbled / resentful*** */* ***content / frustrated*** *by/with how we have been treated so far, and I am of course extremely* ***optimistic / envious / joyful / irritated*** *for/of/with applicants that have been more successful than us for building-related funding. I would like to ask you to consider nominating our application on the online form for special consideration next time - this can remain anonymous if you wish and it would mean we had a higher chance of success with our next funding application for* ***church / mosque*** *repairs.*

*Kind Regards,*

***Alex, on behalf of all at the church /***

***Nour, on behalf of all at the mosque***

**b.**

*Dear resident,*

*I would like to let you know that the* ***Christian / Muslim*** *community centre has recently had some funding cut and because of this we are launching a small business selling freshly cooked traditional cuisine every weekday lunchtime from the kitchen. I feel immensely* ***humbled / resentful / content / angry*** *we are able to/must do this. Other similar catering businesses have recently been started and I am extremely* ***optimistic / envious / joyful / frustrated*** *about/of their high chance at success. I feel great* ***admiration*** */* ***contempt / tenderness / irritation*** *for all small businesses in the current climate and I am writing to ask if you would consider endorsing the business on the* ***Christian / Muslim*** *community centre’s page online. This could be completely anonymous and would likely make a tremendous difference in the project’s initial success.*

*Kind Regards,*

***Sam, on behalf of all at the Christian community centre /***

***Nasim, on behalf of all at the Muslim community centre***

**c.**

*Dear resident,*

*I would like to draw your attention to a recent decision made by the local school to reject our application to hold our weekly* ***Christian / Muslim*** *youth music club in their music room. I feel immense(ly)* ***humble / resentful / tenderness / anger*** *about (the difficulty of) this decision, but/and do not believe the plans would have a direct impact on anyone else as all meetings are outside school hours. I feel great* ***admiration / contempt / contentment / frustration*** *for the way the local school makes decisions and I am extremely* ***optimistic / envious / joyful / irritated*** *for/of/that other clubs (that) gained approval to use the school premises. I would like to ask if you would sign an online petition to encourage the school to reconsider the application on behalf of the* ***Christian / Muslim*** *youth music club? This could be completely anonymous and would likely make a huge difference to the outcome.*

*Kind Regards,*

***Charlie, on behalf of all at the Christian youth music club /***

***Tanveer, on behalf of all at the Muslim youth music club***

**d.**

*Dear resident,*

*I am writing to tell you that we at the local* ***Christian / Muslim*** *art group are planning to enter our photography blog into a national competition. Last year we were just beaten to the shortlist and I felt immensely* ***humble / resentful / content / frustrated*** *about the decision, given the competition. I felt extremely* ***optimistic*** */* ***envious / joyful / angry*** *for/of/towards those that had more success than us. Seeing the quality of entries this year makes me feel great* ***admiration / contempt / tenderness / irritation*** *for the art blogging community. With this in mind, I would like to ask you if you would consider up-voting the* ***Christian / Muslim*** *art group’s blog on the website of entries. This would be completely anonymous and would greatly increase its prominence on the site and our chance of success.*

*Kind regards,*

***Robin, on behalf of all at the Christian art group /***

***Majd, on behalf of all at the Muslim art group***

**Counterbalancing of vignettes for Study 4:**

Each participant saw four emails, one of each emotion condition and from different senders. Half of the participants saw emails from ingroup senders and half saw emails from outgroup senders. Email versions and orders were counterbalanced eight ways such that each emotion condition and each sender appeared in each position (first, second, third and fourth) an equal number of times as shown in Table S3.

**Table S3: Vignette counterbalancing in Study 4.** Columns show version and order of email vignettes that participants saw in each counterbalance condition.

|  | C1 | C2 | C3 | C4 | C5 | C6 | C7 | C8 |
| --- | --- | --- | --- | --- | --- | --- | --- | --- |
| V1 | UH Prosocial Alex /  Nour  (a) | NonUH Prosocial Robin / Majd  (d) | NonUH Antisocial Sam / Nasim  (b) | UH Antisocial Charlie / Tanveer  (c) | UH Prosocial Charlie / Tanveer  (c) | NonUH Prosocial Sam / Nasim  (b) | NonUH Antisocial Robin / Majd  (d) | UH Antisocial Alex /  Nour  (a) |
| V2 | UH Antisocial Sam / Nasim  (b) | NonUH Antisocial Charlie / Tanveer  (c) | UH Prosocial Charlie / Tanveer  (c) | NonUH Prosocial Sam / Nasim  (b) | UH Antisocial Robin / Majd  (d) | NonUH Antisocial Alex / Nour  (a) | UH Prosocial Alex /  Nour  (a) | NonUH Prosocial Robin / Majd  (d) |
| V3 | NonUH Prosocial Charlie / Tanveer  (c) | UH Prosocial Sam / Nasim  (b) | UH Antisocial Robin / Majd  (d) | NonUH Antisocial Alex / Nour  (a) | NonUH Prosocial Alex / Nour  (a) | UH Prosocial Robin / Majd  (d) | UH Antisocial Sam / Nasim  (b) | NonUH Antisocial Charlie / Tanveer  (c) |
| V4 | NonUH Antisocial Robin / Majd  (d) | UH Antisocial Alex /  Nour  (a) | NonUH Prosocial Alex / Nour  (a) | UH Prosocial Robin / Majd  (d) | NonUH Antisocial Sam / Nasim  (b) | UH Antisocial Charlie / Tanveer  (c) | NonUH Prosocial Charlie / Tanveer  (c) | UH Prosocial Sam / Nasim  (b) |

C is counterbalance number and V is vignette presentation order. UH refers to uniquely human emotions. NonUH refers to non-uniquely human emotions, which we also call ‘shared with other animals’. The specific sender participants saw (e.g., Nour / Alex) depended on whether participants were in the ingroup or outgroup condition.

**References**

1. Barrett, L. F., Lindquist, K. A. & Gendron, M. Language as context for the perception of emotion. *Trends Cogn. Sci.* **11**, 327–332 (2007).

2. Sauter, D. A., Eisner, F., Ekman, P. & Scott, S. K. Cross-cultural recognition of basic emotions through nonverbal emotional vocalizations. *Proc. Natl. Acad. Sci.* **107**, 2408–2412 (2010).

3. Cowen, A. S. & Keltner, D. Self-report captures 27 distinct categories of emotion bridged by continuous gradients. *Proc. Natl. Acad. Sci.* **114**, E7900–E7909 (2017).

4. Lewis, M., Haviland-Jones, J. M. & Barrett, L. F. *Handbook of emotions*. (Guilford Press, 2010).

5. Demoulin, S. *et al.* Dimensions of “uniquely” and “non‐uniquely” human emotions. *Cogn. Emot.* **18**, 71–96 (2004).

6. Ekman, P. An argument for basic emotions. *Cogn. Emot.* **6**, 169–200 (1992).

7. Leyens, J.-P. *et al.* Psychological essentialism and the differential attribution of uniquely human emotions to ingroups and outgroups. *Eur. J. Soc. Psychol.* **31**, 395–411 (2001).

8. Paladino, M.-P. *et al.* Differential Association of Uniquely and Non Uniquely Human Emotions with the Ingroup and the Outgroup. *Group Process. Intergroup Relat.* **5**, 105–117 (2002).

9. Cortes, B. P., Demoulin, S., Rodriguez, R. T., Rodriguez, A. P. & Leyens, J.-P. Infrahumanization or Familiarity? Attribution of Uniquely Human Emotions to the Self, the Ingroup, and the Outgroup. *Pers. Soc. Psychol. Bull.* **31**, 243–253 (2005).
